# A better interpretation of data regarding the opioid switching to methadone

**DOI:** 10.1186/s12904-023-01163-y

**Published:** 2023-06-07

**Authors:** Sebastiano Mercadante

**Affiliations:** Main regional center for pain relief and supportive/palliative care, La Maddalena Cancer Center, Via San Lorenzo 312, Palermo, 90145 Italy

**Keywords:** Cancer pain, Methnadone, Opioid switching or rotation, Palliative care

## Abstract

In a recent study methadone has been reported more effective witha 3-day switch (3DS) was more effective than the stop and go strategy (SAG). Many shorcomings, however, are of concerns. The poor selection fo patients with low level of pain intensity, the incomprehensibile choice of of SAG or 3DS, and considerations reported in a previous controlled study with evident methodological limits, make their conclusion inaccurate. Controlled studies are fundamental in research. However, a pragmatic approach reflecting daily practice should be carefully taken into consideration. A more flexible use of SAG strategy and strict clinical observation to change doses according to the clinical response may provide the optimal treatment in patients receiving high doses of opioids.

A retrospetive study of Ding et al. [1] reported that methadone is an effective, safe, and cost saving treatment for patients with refractory cancer pain. This conclusion confirms data of several studies performed in the last 30 years [2]. However, they also report that 3-day switch (3DS) was more effective than the stop and go strategy (SAG). There are many points that are not convincing for making this statement.

First, given that most patients were switched for uncontrolled pain, I wonder if patients were under this condition. In fact the median pain score was 4/10 (presuming that some of them had even less than 4), a level that is not exactly considered a condition of “poor pain control”. A decrease in pain intensity to 2/10 does not seem clinically relevant, although it apparently means a reduction of 50%. Secondly, being a retrospective study, the modality of switching to methadone reflects the practice of the local institution. So, it is difficult to understand why some patients were assigned to 3DS or SAG.

Third, more importantly, they quote a randomized study that revealed that the SAG approach was associated with compromised pain control, a higher number of dropouts, and more serious adverse events than the 3DS approach [3). There are some considerations that could help in making clear some points, regardless of data reported with the strict protocol dictated by the rules of a randomized controlled study.

They do not report how long they needed for achieving the benefit in pain control (regardless of the low level of pain intensity). The rationale of 3DS was based on concerns about the safety of switching to methadone, due to poor information about conversion ratios existing at that time. However, the SAG strategy argues that an unfavorable clinical condition associated with a drug, can be optimally changed, stopping the offending drug and providing a new one. In fact there is no reason to maintain a drug used in more than half of patients at high doses (> 300 mg of oral morphine equivalents (OME)/day). These dose levels are often associated to states of hyperalgesia which suggest to discontinuing the offending drug, rather than providing a certain amount for three days. In addition, the 3DS approach may take time, ranging from 3 to 11 days, which is inconvenient in circumstances of uncontrolled symptoms, resulting in avoidable prolonged suffering.

The SAG strategy has gained popularity for the obvious pharmacokinetic advantages, producing rapid changes of plasma concentration of the two opioids, and consequently of the clinical effects produced by them, rather than facing the effects of two drugs together [3].

But the most relevant point that has been omitted is that while providing a more rapid effect, SAG also requires expertise and flexibility in the subsequent days in managing methadone doses according to the clinical picture, particularly when the switching is started with high opioid doses and requires careful monitoring in a protected environment (such as in the case reported in the Deng’s paper). The rationale of 3DS seems to be already overcome, as there is no advantage in reducing the doses of a drug which is poorly effective or toxic. It is obvious that SAG cannot provide the best performance with a protocol in which doses are not flexible in the subsequent days. In other words SAG requires a high level of experience to carefully monitor the clinical effects, balancing the benefits of pain relief and the occurrence of adverse effects. I reported the many bias of this paper [4]. Norwegian authors reported, for example, that SAG group experienced more adverse effects and drop-outs. Apart from the low number of patients (most patients dropped out for different reasons, not necessarily adverse effects), pain relief in the SAG group seems to be lower at day 3 [5]. Patients who develop adverse effects are likely to be overdosed, if methadone concentrations are presumed to be higher in the SAG approach. As a consequence, one could also expect a marked analgesia. Of interest, the only difference between SAG and 3DS groups resides in the first two days when more rescue doses per day are given in the 3DS group. Methadone dosage was then similar from the 3rd day to the 14th day, with patients receiving a similar treatment whether they use methadone as a rescue drug (this data is lacking). Thus the differences reported in pain intensity at day 14 are unexplainable as patients in both groups are receiving the same treatment for more than 10 days. Ten patients of SAG group completed the study and were compared with 18 patients who used 3DS, with obvious statistical implications. Data, however, are inferred by non assuming that SAG strategy requires flexibility in dosing, rather than maintaining the same doses as per protocol. While controlled studies are fundamental in research, a pragmatic approach reflecting daily practice should be carefully taken into consideration [6]. The same Norwegian group concluded that to stop the previous opioid, and under expert supervision, to change the doses of methadone according to the clinical response might be the best choice [7].

While we agree that methadone is an optimal and cost-effective choice for patients who do not respond to previous opioids, a more flexible use of SAG strategy and strict clinical observation to change doses according to the clinical response may provide the optimal treatment in patients receiving high doses of opioids. Retrospective comparison studies have relevant limitations and data gathered from this kind of study may be misleading and requires careful interpretation.

## Data Availability

NA.
